# 

**Published:** 1875-02

**Authors:** 


Vol. 22. FEBRUARY, 1875. No. 2.

TWENTY-SECOND YEAR.

### HALL'S Journal of Health.

PUBLISHED BY THE AMERICAN AND FOREIGN PUBLICATION CO., 137 EIGHTH ST., NEW YORK.

Agents for the Trade:

AMERICAN NEWS COMPANY, 121 NASSAU STREET.
NEW YORK NEWS COMPANY, 18 BEEKMAN STREET.

Terms, Two Dollars a Year.
SINGLE NUMBER, 20 CENTS.

